# Genome sequences of copper resistant and sensitive *Enterococcus faecalis* strains isolated from copper-fed pigs in Denmark

**DOI:** 10.1186/s40793-015-0021-1

**Published:** 2015-07-08

**Authors:** Siyu Zhang, Dan Wang, Yihua Wang, Henrik Hasman, Frank M. Aarestrup, Hend A. Alwathnani, Yong-Guan Zhu, Christopher Rensing

**Affiliations:** 1 Department of Plant and Environmental Science, University of Copenhagen, Frederiksberg, Denmark; 2 State Key Laboratory of Urban and Regional Ecology, Research Center for Eco-Environmental Sciences, Chinese Academy of Sciences, Beijing, China; 3 State Key Laboratory of Agricultural Microbiology, College of Life Sciences and Technology, HuaZhong Agricultural University, Wuhan, China; 4 National Food Institute, Technical University of Denmark, Kgs. Lyngby, Denmark; 5 Department of Botany and Microbiology, King Saud University, Riyadh, Saudi Arabia; 6 Key Laboratory of Urban Environment and Health, Institute of Urban Environment, Chinese Academy of Sciences, Xiamen, China

**Keywords:** Enterococcus faecalis, Copper resistance, Antibiotic resistance, Genome sequence, Comparative genomics

## Abstract

Six strains of *Enterococcus faecalis* (S1, S12, S17, S18, S19 and S32) were isolated from copper fed pigs in Denmark. These Gram-positive bacteria within the genus *Enterococcus* are able to survive a variety of physical and chemical challenges by the acquisition of diverse genetic elements. The genome of strains S1, S12, S17, S18, S19 and S32 contained 2,615, 2,769, 2,625, 2,804, 2,853 and 2,935 protein-coding genes, with 41, 42, 27, 42, 32 and 44 genes encoding antibiotic and metal resistance, respectively. Differences between Cu resistant and sensitive *E. faecalis* strains, and possible co-transfer of Cu and antibiotic resistance determinants were detected through comparative genome analysis.

## Introduction

Copper is an essential trace element with an ubiquitous cellular distribution and performs several biological functions [1]. It serves as an important structural component or catalytic co-factor for a wide range of different enzymes in various important biochemical pathways in bacteria, plants and animals [2]. Because Cu, among many other micronutrients, is beneficial for growth promotion and feed efficiency of farm animals [3], [4], it is extensively used as an additive in swine feed. Normally, the concentration of Cu used in animal feed is in excess of the nutritional requirements of animals as it is used as an alternative to in-feed antibiotics for prevention of diarrheal disease [5]. Therefore, enteric bacteria, both commensal and pathogenic, in these animals have typically acquired several additional Cu resistance determinants to survive its toxicity [1], [6], [7].

*Enterococci* belong to the gastrointestinal flora of humans and animals, and have been known for more than a century for their pathogenicity to humans, causing urinary tract and surgical wound infections, bacteraemia and endocarditis [8]. Currently, more than 30 species within the genus *Enterococcus * have been described, and the two most studied enterococcal species are *Enterococcus faecium * and *Enterococcus faecalis *[9]. Over the last two decades, *E. faecalis * and *E. faecium * have become increasingly important nosocomial pathogens worldwide and are difficult treat due to their increasing multidrug resistance [10]. The intrinsic resistance of *Enterococcus * to many antibiotics and its acquisition of resistance determinants to other antimicrobial agents led to the emergence of *Enterococcus * as a nosocomial pathogen [11], [12]. Recently, the co-selection of MDR isolates by antibiotics, metals and biocides has been reported [13], [14], and the resistance of *Enterococcus * to both Cu and antibiotics has been established [15], [16]. However, few studies have addressed gene transfer and the underlying molecular mechanisms of the various Cu resistance determinants in *E. faecalis *[17]. Herein, we present the genome sequences along with the main features of six *E. faecalis * strains showing the differences between Cu resistant and sensitive strains of *E. faecalis *, and suggesting possible co-transfer of Cu and antibiotic resistance determinants in these bacteria.

## Organism information

### Classification and Features

**Fig. 1:**
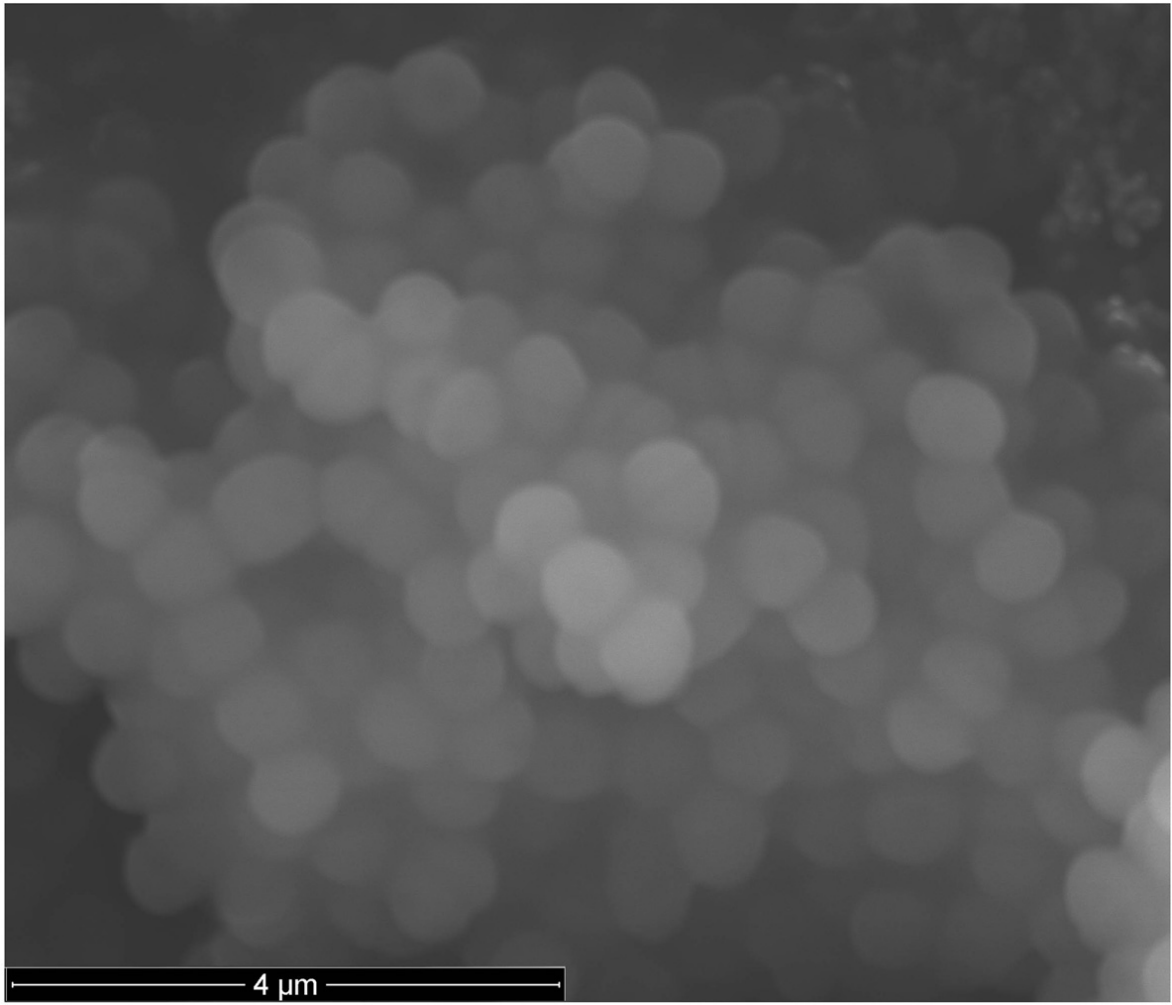
Phylogenetic tree highlighting the position of the six *E. faecalis* strains relative to phylogenetically closely related type strains within the genus *Enterococcus*. The sequences were aligned using Clustal W, and the neighbor-joining tree was constructed based on kimura 2-parameter distance model using MEGA 6.0. Bootstrap values above 50 % are shown obtained from 1,000 bootstrap replications. Bar, 0.02 substitutions per nucleotide position. GenBank accession numbers are displayed in parentheses. Large triangles represent the six *Enterococcus* strains sequenced in this study

Phylogenetic analysis was performed using the 16S rRNA gene sequences on the six strains S1, S12, S17, S18, S19 and S32 and related species. Sequences were aligned using Clustal W, and a phylogenetic tree was constructed using neighbor-joining (NJ) method implemented in MEGA version 6.0. The resultant tree topologies were evaluated by bootstrap analyses with 1,000 random samplings. Phylogenetic analysis based on 16S rRNA gene sequences showed that the six strains clustered together with *E. faecalis *ATCC 29212 and *E. faecalis * SFL with a high bootstrap value (100 %). All the *E. faecalis * are in a distinct branch with the other enterococci, such as *E. casseliflavus *, *E. faecium *, *E. hirae * and the another pig gut *Firmicute*, that is *Streptococcus equinus * NCDO 1037 (Fig. 1). The six strains could be classified as members of the genus *Enterococcus * based on their 16S rRNA gene phylogeny and phenotypic characteristics (Table 1).

**Table 1:** Classification and general features of the six Enterococcus faecalis strains according to the MIGS recommendations [26]

MIGS ID	Property	Term	Evidence code ^a^
	Current classification	Domain: *Bacteria*	TAS [38]
		Phylum: *Firmicutes*	TAS [39]
		Class: *Bacilli*	TAS [40]
		Order: *Lactobacillales*	TAS [41]
		Family: *Enterococcaceae*	TAS [42]
		Genus: *Enterococcus*	TAS [18], [19]
		Species: *Enterococcus faecalis*	TAS [43]
		Strain: S1, S12, S17, S18, S19, S32	NAS
	Gram stain	Positive	TAS [42]
	Cell shape	Oval cocci	TAS [42]
	Motility	None	TAS [44]
	Sporulation	Non-sporulating	TAS [43]
	Temperature range	10-45 °C	TAS [22]
	Optimum temperature	37 °C	TAS [22]
	pH range	4.6-9.9 (Optimum pH at 7.5)	TAS [22]
MIGS-6	Habitat	Gastrointestinal tracts of humans and other mammals	TAS [8]
MIGS-6.3	Salinity	0-6.5 %	TAS [22]
MIGS-22	Oxygen	Facultatively anaerobic	TAS [44]
MIGS-15	Biotic relationship	Commensal bacterium	TAS [8]
MIGS-14	Pathogenicity	Highly pathogenic	TAS [43]
MIGS-4	Geographic location	Denmark	NAS
MIGS-5	Sample collection	2011	NAS
MIGS-4.1	Latitude	Unknown	NAS
MIGS-4.2	Longitude	Unknown	NAS
MIGS-4.3	Altitude	Unknown	NAS

^a^ Evidence codes - TAS: Traceable Author Statement (i.e., a direct exists in the literature); NAS: Non-traceable Author Statement (i.e., not directly observed for the living, isolated sample, but based on a generally accepted property for the species, or anecdotal evidence). These evidence codes are from the Gene Ontology project [45]

**Fig. 2:**
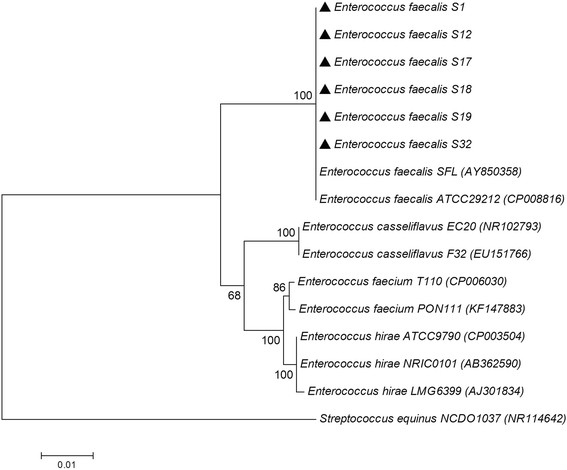
Micrograph of *E. faecalis* strains obtained by scanning electron microscopy. Scale bar, 4 m

*E. faecalis * is a Gram-positive, oval-shaped, and often highly pathogenic bacterium classified as a member of the genus *Enterococcus * (Table 1 and Fig. 2) [18], [19]. It is a natural inhabitant of the mammalian gastrointestinal tract and is commonly found in soil, sewage, water and food [8]. *E. faecalis * is quite versatile and able to survive a variety of physical and chemical challenges by the acquisition of diverse genetic elements, which may contribute to their adaption to different hosts and environments [20], [21]. They are able to grow in temperatures ranging from 0 °C up to 50 °C, and can survive in the presence of 6.5 % NaCl and in broth at pH 9.6 [22]. They can also be resistant to heavy and transition metals [17], as well as many different antibiotics [23]–[25], especially vancomycin [20], [21].

## Genome sequencing information

### Genome project history

The *E. faecalis * strains (S1, S12, S17, S18, S19 and S32) were isolated from Cu-fed pigs as part of the Danish Integrated Antimicrobial Resistance Monitoring (DANMAP) surveillance program [23]. The isolates were collected from healthy animals at or just prior to slaughter. Those whole-genome shotgun projects have been deposited in DDBJ/EMBL/GenBank under the accession number JTKS00000000, JTKT00000000, JTKU00000000, JTKV00000000, JTKW00000000 and JTKX00000000. Table 2 presents the project information and its association with MIGS version 2.0 compliance [26]. Cu resistant strains are *E. faecalis * strains S1, S18, S32, while the other three strains are Cu sensitive.

**Table 2:** Project information

MIGS ID	Property	Term/Strains					
		S1	S12	S17	S18	S19	S32
MIGS-31	Finishing quality	High-quality draft					
MIGS-28	Libraries used	One paired-end Illumina library					
MIGS-29	Sequencing platforms	Illumina Miseq					
MIGS-31.2	Fold coverage	156	162	240	84	172	200
MIGS-30	Assemblers	Velvet version 1.1.04					
MIGS-32	Gene calling method	Glimmer 3.0					
	Genbank ID	JTKS00000000	JTKT00000000	JTKU00000000	JTKV00000000	JTKW00000000	JTKX00000000
	Genbank Date of Release	2014/12/02					
	Bioproject	PRJNA267758	PRJNA268957	PRJNA268240	PRJNA268137	PRJNA267759	PRJNA268241
	Project relevance	Environmental					
MIGS-13	Source Material Identifier	Strain: 1	Strain: 12	Strain: 17	Strain: 18	Strain: 19	Strain: 32
	Project relevance	Environment, bacteria isolated from copper fed pigs					

Copper resistant strains are marked in red (S1, S18 and S32)

### Growth conditions and genomic DNA preparation

*E. faecalis * were streaked on Slanetz agar (BD Difco) plates and grown for 48 h at 42 °C. Each strain was inoculated separately into 25 ml of brain heart infusion broth at 37 °C for 24 h. Genomic DNA was purified from the isolates using the Easy-DNA extraction kit (Invitrogen), and DNA concentrations were determined by the Qubit dsDNA BR assay kit (Invitrogen).

### Genome sequencing and assembly

Whole genome sequencing of *E. faecalis * strains S1, S12, S17, S18, S19 and S32 was carried out on an Illumina Miseq platform (Illumina, Inc., San Diego, CA). Genomic libraries were prepared by the Nextera XT DNA sample preparation kit (Illumina, cat. No. FC-131-1024), and then sequenced using v3, 2 × 300 bp chemistry on the Illumina MiSeq platform. Genomic assemblies were constructed using Velvet version 1.1.04, generating 24, 57, 20, 103, 34 and 89 contigs, respectively.

## Genome annotation

The resulting contigs were uploaded onto the Rapid Annotation using Subsystem Technology server databases and the gene-caller GLIMMER 3.02 [27], [28] to predict open reading frames. The predicted ORFs were translated and annotated by searching against clusters of orthologous groups using the SEED databases [29], as well as NCBI databases. RNAmmer 1.2 [30] and tRNAscan SE 1.23 [31] were used to identify rRNA genes and tRNA genes, respectively. CRISPR repeats were examined using CRISPR recognition tool (CRT) [32].

## Genome properties

**Fig. 3:**
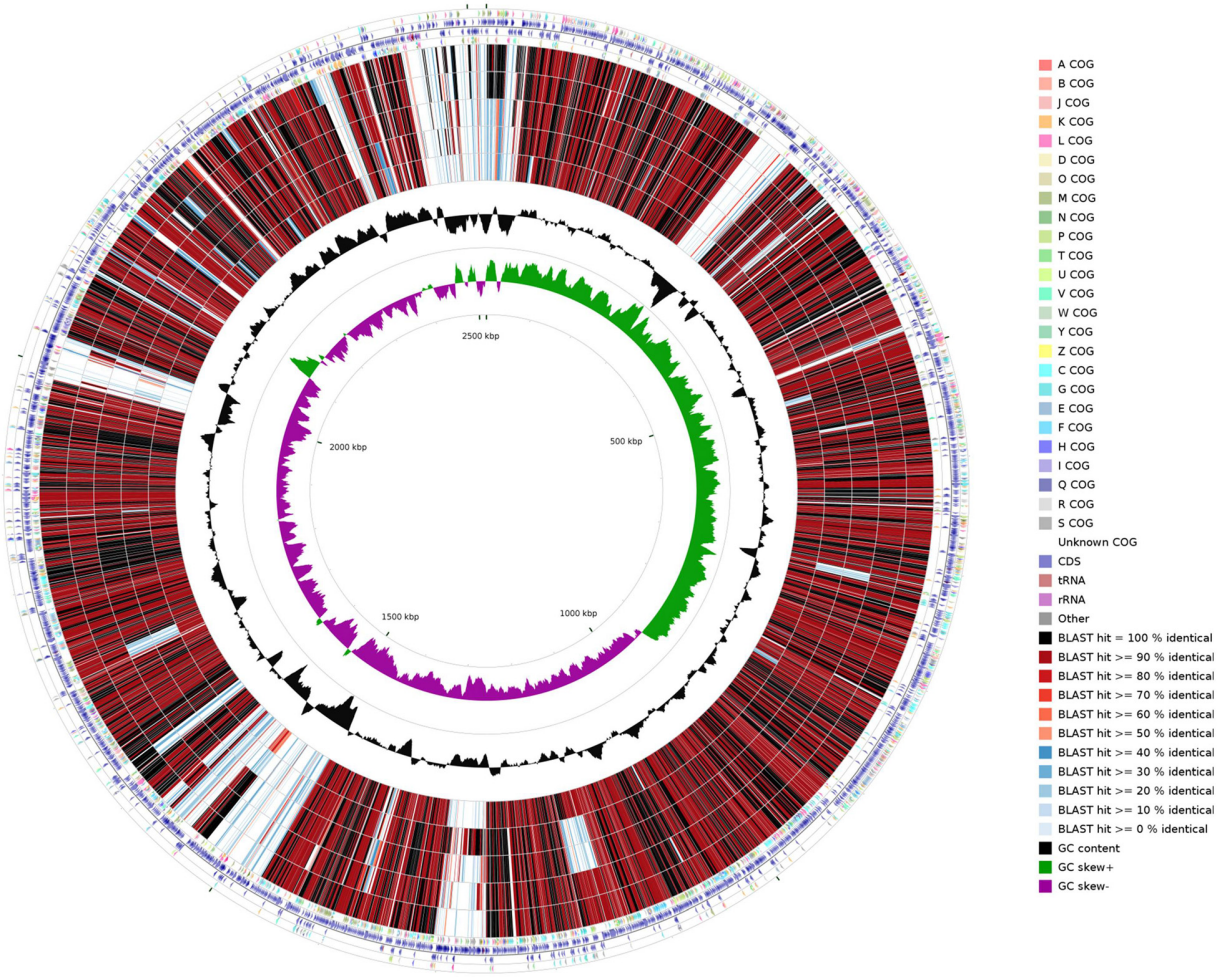
Graphical circular map of the genome comparison of *E. faecalis* S32 with the other five strains. Labeling from the outside to the inside circle: ring 1 and 4 show the protein coding genes on the forward/reverse strand (colored by COG categories); ring 2 and 3 show the denote genes on the forward/reverse strand; ring 5, 6, 7, 8 and 9 show the CDS vs CDS BLAST results of *E. faecalis* S32 with S1, S18, S12, S19 and S17, respectively; ring 10 shows the G + C content (peaks out/inside the circle indicate values higher or lower than the average G + C content, respectively); ring 11 shows GC skew (calculated as (G - C)/(G + C), peaks out/inside the circle indicates values higher or lower than 1, respectively). Ring 5–9 were arranged based on the CDS BLAST results, with the similarity rank from high to low, that is S1 and S18 were more similar to the reference strain S32 than the other three strains

Whole genome sequencing of *E. faecalis * strains S1, S12, S17, S18, S19 and S32 resulted in 156, 162, 240, 84, 172 and 200 fold coverage of the genomes, respectively. The draft genome sizes were 2,762,808, 2,896,725, 2,786,673, 2,888,656, 2,969,229 and 3,037,709 bp in length, with an average GC content of 37.6, 37.4, 37.5, 37.4, 37.2 and 37.2 %, respectively, and comprises 2,615; 2,769; 2,625; 2,804; 2,853 and 2,935 protein coding sequences, respectively. Of the protein coding genes, 2,002; 2,006; 1,949; 2,001; 2,058 and 2,073 were genes with function predictions, with 41, 42, 27, 42, 32 and 44 genes responsible for antibiotics and toxic compounds resistant, respectively. There are 52 (4 rRNA genes and 48 tRNA genes), 54 (3 rRNA genes and 51 tRNA genes), 48 (3 rRNA genes and 45 tRNA genes), 52 (4 rRNA genes and 48 tRNA genes), 53 (3 rRNA genes and 50 tRNA genes) and 55 (5 rRNA genes and 50 tRNA genes) RNA genes for strains S1, S12, S17, S18, S19 and S32, respectively. The properties and statistics for the genome are summarized in Table 3. The distribution of genes into COG functional categories is presented in Table 4 and Fig. 3.

**Table 3:** Genome statistics

Attribute	Strain											
	S1		S12		S17		S18		S19		S32	
	Value	%	Value	%	Value	%	Value	%	Value	%	Value	%
Contigs	24	-	57	-	20	-	103	-	34	-	89	-
Genome size (bp)	2,762,808	100	2,896,725	100	2,786,673	100	2,888,656	100	2,969,229	100	3,037,709	100
DNA coding region (bp)	2,443,661	88.45	2,539,142	87.66	2,451,937	87.99	2,539,829	87.92	2,579,002	86.86	2,639,903	86.90
DNA G + C content (bp)	1,038,816	37.6	1,083,375	37.4	1,045,002	37.5	1,080,357	37.4	1,104,553	37.2	1,130,028	37.2
Total genes	2,701	100	2,864	100	2,706	100	2,892	100	2,962	100	3,043	100
Protein-coding genes	2,615	98.09	2,769	98.09	2,625	98.21	2,804	98.15	2,853	98.15	2,935	98.17
RNA genes	52	1.93	54	1.89	48	1.77	52	1.80	53	1.79	55	1.81
Pseudo genes	35	1.30	43	1.50	34	1.26	36	1.24	59	1.99	63	2.07
Genes in internal clusters	1,150	42.58	1,228	42.88	1,127	41.65	1,256	43.43	1,265	42.71	1,313	43.15
Genes with function prediction	2,002	76.56	2,006	72.44	1,949	74.25	2,001	71.36	2,058	72.13	2,073	70.63
Genes assigned to COGs	2,011	76.90	2,024	73.09	1,980	75.43	2,025	72.22	2,049	71.82	2,084	71.01
Genes with Pfam domains	2,268	86.73	2,313	83.53	2,231	84.99	2,282	81.38	2,318	81.25	2,374	80.89
Genes with signal peptides	575	21.99	614	22.17	600	22.86	590	21.04	632	22.15	639	21.77
Genes with transmembrane helices	729	27.88	769	27.77	756	28.80	754	26.89	779	27.30	797	27.16
CRISPR repeats	1	-	1	-	2	-	1	-	2	-	1	-

The total is based on either the size of the genome in base pairs or the total number of protein coding genes in the annotated genome

**Table 4:** Number of genes associated with the 25 general COG functional categories

Code	Attribute	Strain											
		S1		S12		S17		S18		S19		S32	
		Value	%	Value	%	Value	%	Value	%	Value	%	Value	%
J	Translation, ribosomal structure and biogenesis	155	5.93	152	5.49	152	5.79	153	5.46	152	5.33	153	5.21
A	RNA processing and modification	-	-	-	-	-	-	-	-	-	-	-	-
K	Transcription	172	6.58	178	6.43	174	6.63	173	6.17	183	6.41	184	6.27
L	Replication, recombination and repair	114	4.36	125	4.51	112	4.27	127	4.53	127	4.45	132	4.50
B	Chromatin structure and dynamics	-	-	-	-	-	-	-	-	-	-	-	-
D	Cell cycle control, mitosis and meiosis	22	0.84	25	0.90	22	0.84	21	0.75	23	0.81	24	0.82
Y	Nuclear structure	-	-	-	-	-	-	-	-	-	-	-	-
V	Defense mechanisms	56	2.14	45	1.63	51	1.94	46	1.64	46	1.61	54	1.84
T	Signal transduction mechanisms	90	3.44	89	3.21	85	3.24	94	3.35	87	3.05	95	3.24
M	Cell wall/membrane biogenesis	105	4.02	100	3.61	107	4.08	105	3.74	98	3.43	123	4.19
N	Cell motility	10	0.38	10	0.36	11	0.42	9	0.32	12	0.42	12	0.41
Z	Cytoskeleton	-	-	-	-	-	-	-	-	-	-	-	-
W	Extracellular structures	-	-	-	-	-	-	-	-	-	-	-	-
U	Intracellular trafficking and secretion	24	0.92	25	0.90	25	0.95	27	0.96	24	0.84	24	0.82
O	Posttranslational modification, protein turnover and chaperons	50	1.91	49	1.77	48	1.83	48	1.71	49	1.72	48	1.64
C	Energy production and conversion	106	4.05	106	3.83	105	4.00	106	3.78	107	3.75	106	3.61
G	Carbohydrate transport and metabolism	269	10.29	282	10.18	264	10.06	262	9.34	296	10.38	277	9.44
E	Amino acid transport and metabolism	173	6.62	172	6.21	169	6.44	176	6.28	171	5.99	173	5.89
F	Nucleotide transport and metabolism	93	3.56	90	3.25	87	3.31	93	3.32	92	3.22	90	3.07
H	Coenzyme transport and metabolism	69	2.64	68	2.46	68	2.59	72	2.57	66	2.31	72	2.45
I	Lipid transport and metabolism	56	2.14	56	2.02	57	2.17	59	2.10	56	1.96	58	1.98
P	Inorganic ion transport and metabolism	118	4.51	115	4.15	110	4.19	119	4.24	112	3.93	115	3.92
Q	Secondary metabolism biosynthesis, tansport and catabolism	28	1.07	28	1.01	28	1.07	31	1.11	27	0.95	30	1.02
R	General function prediction only	249	9.52	251	9.06	245	9.33	255	9.09	253	8.87	253	8.62
S	Function unknown	218	8.34	224	8.09	222	8.46	220	7.85	235	8.24	238	8.11
-	Not in COGs	604	23.10	745	26.91	645	24.57	779	27.78	804	28.18	851	28.99

The total is based on the total number of protein coding genes in the annotated genome

## Insights from the genome sequence

All of the six strains contain a four gene operon, *copYZAB,* encoding a Cu resistance determinant (Table 5), which was initially observed in the Gram-positive bacterium *E. hirae *[33]. CopA and CopB are P-type ATPases responsible for ATP-dependent Cu ^+^ transport across the cytoplasmic membranes. The Cu chaperone CopZ binds two Cu ^+^ atoms in a solvent accessible manner, presumably to facilitate their transfer to the transcriptional regulator CopY. Upon binding Cu ^+^ , CopY undergoes a conformational change and is released from the *copA* operator allowing expression of the *copYZAB* operon [1]. A gene encoding the cytoplasmic Cu homeostasis protein CutC was identified in all six strains (Table 5), and CutC has been demonstrated to be involved in Cu homeostasis in *E. faecalis *[34]. In addition, another possible gene encoding a putative Cu ^+^ -translocating P-type ATPase, was identified in all six strains named *ctpA* in this study (Table 5). The genome comparisons of the six *E. faecalis * strains using *E. faecalis * S32 as the reference strain by CGview comparison tool [35] indicated that S1 and S18 were more similar to the reference strain S32 than the other three strains (Fig. 3).

**Table 5:** Copper and antibiotic resistance genes in *E. faecalis* strains. S1, S18 and S32 represent the three Cu resistant *E. faecalis* strains, and S12, S17 and S19 represent the three Cu sensitive *E. faecalis* strains

Genes	Strain name					
	S1	S18	S32	S12	S17	S19
*copY*	++	++	++	+	+	+
*copA*	+	+	+	+	+	+
*copB*	+	+	+	+	+	+
*copZ*	+	+	+	+	+	+
*tcrY*	+	+	+	–	–	–
*tcrA*	+	+	+	–	–	–
*tcrB*	+	+	+	–	–	–
*tcrZ*	+	+	+	+	–	–
*ctpA*	+	+	+	+	+	+
*cueO*	+	+	+	–	–	–
*cutC*	+	+	+	+	+	+
*tetM*	+	+	+	+	–	–
*vanA*	–	–	+	–	–	–
Streptothricin acetyltransferase gene	+	+	+	–	–	–
Aminoglycoside adenylyltransferase gene	+	+	–	–	–	–

*copYABZ* copper resistance genes in sensitive strains (For S1, S18 and S32, one of the *copY* is on the Cu resistant island, and the other is on the chromosome.); *tcrYABZ* copper resistance genes in resistant strains; *ctpA*: copper resistance genes; *cueO*: multicopper oxidase genes; *cutC*: genes encoding cytoplasmic copper homeostasis protein; *tetM*: tetracycline resistance genes; *vanA*: vancomycin resistance genes; Streptothricin acetyltransferase gene: streptothricin resistance genes

**Fig. 4:**
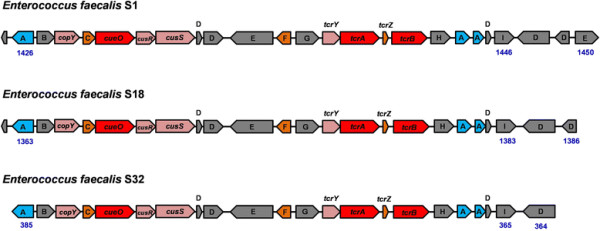
Cu pathogenicity island in *E. faecalis* S1, S18 and S32. **a**: prolipoprotein diacylglyceryl trPropertyansferase, **b**: intergral membrane protein, **c**: chaperone, **d**: hypothetical protein, **e**:transposase, **f**: disrupted P-type ATPase, **g**: integrase, **h**: adenylate kinase, **i**: resolvase, *copY*: CopY family transcriptional regulator, *cueO*: multicopper oxidase, *cusR*: Cu(I)-sensing regulator, *cusS*: Cu(I)-sensing sensor, *tcrY*: *tcrYAZB* operon regulator, *tcrA*: putative copper-efflux CPx-type ATPase, *tcrB*: Cu ^+^ -translocating CPx-type ATPase, *tcrZ*: putative chaperone

The *tcrYAZB* operon was initially identified on the pA17sv1 plasmid in *E. faecium *, which also carried genes encoding resistance to erythromycin (*ermB*) and vancomycin (*vanA*) [17], [36]. High toxic Cu levels could be tolerated due to the presence of *tcrB* in *E. faecium * or *E. faecalis * which encodes a Cu ^+^ -translocating P-type ATPase homologous to CopB encoded on *copYZAB* operon [37]. Comparing these six *E. faecalis * strains against others previously identified with increased Cu resistance, the *tcrYAZB* operon and adjacent *cueO* encoding a multicopper oxidase were only identified in *E. faecalis * S1, S18 and S32 (Table 5). Blasting of the *tcrYAZB* operon against the contigs of the other three strains verified that they were indeed lacking Cu resistance genes. The *cueO* gene identified in putative copper resistant strains encodes a multicopper oxidase that is transported across the cytoplasmic membrane and oxidizes Cu(I) to Cu(II) and so aids protection from high Cu concentrations in *Enterococcus *[9] or other Gram-positive strains [16]. The approximate 20-gene copper pathogenicity/fitness island present in *E. faecalis * S1, S18 and S32, show *cueO* is located in close vicinity of *tcrYAZB* and probably regulated by an adjacent two-component regulator system (Cu(I)-sensing regulator (*cusR*) and Cu(I)-sensing sensor (*cusS*)) (Fig. 4). Transposase and mobile element protein genes were also identified on this pathogenicity/fitness island next to *tcrYAZB*, indicating mobility. Moreover, genes encoding prolipoprotein diacylglyceryl transferase, which is responsible for oxidative stress tolerance potentially also caused by Cu ^+^ , could be identified on these potential pathogenicity and/or fitness islands as well. For the other three Cu sensitive *E. faecalis * S12, S17 and S19, *tcrYAZB*, *cueO*, *cusR*, *cusS* or genes encoding a prolipoprotein diacylglyceryl transferase could not be detected.

The antibiotic resistance gene *tetM* (resistance to tetracycline) could be identified in the three Cu resistant *E. faecalis * S1, S18, S32, and Cu sensitive *E. faecalis * S12; *vanA* (encoding vancomycin resistance) was identified only in Cu resistant *E. faecalis * S32; streptothricin acetyltransferase gene was identified in the Cu resistant *E. faecalis * S1, S18, S32; and aminoglycoside adenylyltransferase gene was identified in two Cu resistant *E. faecalis * S1 and S18 (Table 5).

## Conclusions

Since the co-transfer of genes encoding antibiotic resistance along with Cu tolerance genes in one transconjugant has been demonstrated [14], the results in this study might provide valuable information corroborating the co-transfer of genes encoding additional Cu resistance and genes encoding numerous antibiotic resistances. Also, the identified antibiotic resistance gene *tetM* in all the Cu resistant strains is consistent with the MDR *Enterococcus * strains observed in the environment [13]–[16].

## Abbreviation

MDR: Multidrug-resistant

## Competing interests

The authors declare that they have no competing interests.

## Authors’ contributions

SZ drafted the manuscript, performed laboratory experiments, and analyzed the data. DW and YW performed the comparative genome analysis. HH, FA and HA sequenced, assembled, and annotated the genome. YZ revised the manuscript. CR organized the study and revised the manuscript. All authors read and approved the final manuscript.

## References

[B1] Samanovic MI Ding C Thiele DJ Darwin KH Copper in microbial pathogenesis: meddling with the metal Cell Host Microbe 2012 11 2 106 15 10.1016/j.chom.2012.01.009 22341460 PMC3285254

[B2] Yazdankhah S, Rudi K, Bernhoft A. Zinc and copper in animal feed–development of resistance and co-resistance to antimicrobial agents in bacteria of animal origin. Microb Ecol Health Dis. 2014;25. 10.3402/mehd.v25.25862 PMC4179321 25317117

[B3] Cunha T. Swine feeding and nutrition. New York: Elsevier; 2012.

[B4] Jacob ME Fox JT Nagaraja T Drouillard JS Amachawadi RG Narayanan SK Effects of feeding elevated concentrations of copper and zinc on the antimicrobial susceptibilities of fecal bacteria in feedlot cattle Foodborne Pathogens Dis 2010 7 6 643 8 10.1089/fpd.2009.0401 20482227

[B5] Monteiro SC Lofts S Boxall A Pre-assessment of environmental impact of zinc and copper used in animal nutrition 2010

[B6] Hodgkinson V Petris MJ Copper homeostasis at the host-pathogen interface J Biol Chem 2012 287 17 13549 55 10.1074/jbc.R111.316406 22389498 PMC3340201

[B7] Amachawadi R Shelton N Shi X Vinasco J Dritz S Tokach M Selection of fecal enterococci exhibiting *tcrB*-mediated copper resistance in pigs fed diets supplemented with copper Appl Env Microbiol 2011 77 16 5597 603 10.1128/AEM.00364-11 21705534 PMC3165251

[B8] Murray BE The life and times of the *Enterococcus* Clin Microbiol Rev 1990 3 1 46 65 2404568 10.1128/cmr.3.1.46 PMC358140

[B9] van Schaik W Top J Riley DR Boekhorst J Vrijenhoek JE Schapendonk CM Pyrosequencing-based comparative genome analysis of the nosocomial pathogen *Enterococcus faecium* and identification of a large transferable pathogenicity island BMC Genomics 2010 11 1 239 10.1186/1471-2164-11-239 20398277 PMC2858755

[B10] Willems RJ Top J van Schaik W Leavis H Bonten M Sirén J Restricted gene flow among hospital subpopulations of *Enterococcus faecium* Mbio 2012 3 4 e00151 00112 10.1128/mBio.00151-12 22807567 PMC3413404

[B11] Paulsen I Banerjei L Myers G Nelson K Seshadri R Read T Role of mobile DNA in the evolution of vancomycin-resistant *Enterococcus faecalis* Science 2003 299 5615 2071 4 10.1126/science.1080613 12663927

[B12] de Regt MJ van Schaik W van Luit-Asbroek M Dekker HA van Duijkeren E Koning CJ Hospital and community ampicillin-resistant *Enterococcus faecium* are evolutionarily closely linked but have diversified through niche adaptation PLoS One 2012 7 2 1 9 10.1371/journal.pone.0030319 PMC3281830 22363425

[B13] Novais C, Freitas AR, Silveira E, Antunes P, Silva R, Coque TM, et al. Spread of multidrug-resistant *Enterococcus* to animals and humans: an underestimated role for the pig farm environment. J Antimicrob Chemother. 2013;1–9. 10.1093/jac/dkt289 23861310

[B14] Silveira E Freitas AR Antunes P Barros M Campos J Coque TM Co-transfer of resistance to high concentrations of copper and first-line antibiotics among *Enterococcus* from different origins (humans, animals, the environment and foods) and clonal lineages J Antimicrob Chemother 2014 69 4 899 906 10.1093/jac/dkt479 24343895

[B15] Hasman H Kempf I Chidaine B Cariolet R Ersbøll AK Houe H Copper resistance in *Enterococcus faecium*, mediated by the *tcrB* gene, is selected by supplementation of pig feed with copper sulfate Appl Environ Microbiol 2006 72 9 5784 9 10.1128/AEM.02979-05 16957194 PMC1563648

[B16] Solioz M Abicht HK Mermod M Mancini S Response of Gram-positive bacteria to copper stress JBIC J Biological Inorganic Chem 2010 15 1 3 14 10.1007/s00775-009-0588-3 19774401

[B17] Hasman H The tcrB gene is part of the *tcrYAZB* operon conferring copper resistance in *Enterococcus faecium* and *Enterococcus faecalis* Microbiol 2005 151 9 3019 25 10.1099/mic.0.28109-0 16151212

[B18] Schleifer K, Kraus J, Dvorak C, Kilpper-Bälz R, Collins M, Fischer W. Transfer of *Streptococcus lactis* and related streptococci to the genus *Lactococcus* gen. nov. Syst Appl Microbiol. 1985;6(2):183–95.

[B19] Devriese L Baele M Butaye P The genus *Enterococcus* The Prokaryotes: Volume 4: Bacteria: Firmicutes, Cyanobacteria 2006 163 74

[B20] Arias CA Murray BE The rise of the *Enterococcus:* beyond vancomycin resistance Nat Rev Microbiol 2012 10 4 266 78 10.1038/nrmicro2761 22421879 PMC3621121

[B21] Cattoir V Leclercq R Twenty-five years of shared life with vancomycin-resistant enterococci: is it time to divorce? J Antimicrob Chemother 2013 68 4 731 42 10.1093/jac/dks469 23208830

[B22] Gardini F Martuscelli M Caruso MC Galgano F Crudele MA Favati F Effects of pH, temperature and NaCl concentration on the growth kinetics, proteolytic activity and biogenic amine production of *Enterococcus faecalis* Int J Food Microbiol 2001 64 1 105 17 10.1016/S0168-1605(00)00445-1 11252492

[B23] Use of antimicrobial agents and the occurrence of antimicrobial resistance in bacteria from food animals, foods and humans in Denmark 2005

[B24] Mazaheri Nezhad Fard R Heuzenroeder MW Barton MD Antimicrobial and heavy metal resistance in commensal enterococci isolated from pigs Vet Microbiol 2011 148 2 276 82 10.1016/j.vetmic.2010.09.002 20951513

[B25] Kim J Lee S Choi S Copper resistance and its relationship to erythromycin resistance in *Enterococcus* isolates from bovine milk samples in Korea J Microbiol 2012 50 3 540 3 10.1007/s12275-012-1579-6 22752920

[B26] Field D Garrity G Gray T Morrison N Selengut J Sterk P The minimum information about a genome sequence (MIGS) specification Nat Biotechnol 2008 26 5 541 7 10.1038/nbt1360 18464787 PMC2409278

[B27] Aziz RK Bartels D Best AA DeJongh M Disz T Edwards RA The RAST Server: rapid annotations using subsystems technology BMC Genomics 2008 9 1 75 10.1186/1471-2164-9-75 18261238 PMC2265698

[B28] Meyer F Paarmann D D'Souza M Olson R Glass EM Kubal M The metagenomics RAST server–a public resource for the automatic phylogenetic and functional analysis of metagenomes BMC Bioinformatics 2008 9 1 386 10.1186/1471-2105-9-386 18803844 PMC2563014

[B29] Hemmerich C Buechlein A Podicheti R Revanna KV Dong Q An Ergatis-based prokaryotic genome annotation web server BMC Bioinformatics 2010 26 8 1122 4 10.1093/bioinformatics/btq090 20194626

[B30] Lagesen K Hallin P Rødland EA Stærfeldt H-H Rognes T Ussery DW RNAmmer: consistent and rapid annotation of ribosomal RNA genes Nucleic Acids Res 2007 35 9 3100 8 10.1093/nar/gkm160 17452365 PMC1888812

[B31] Lowe TM Eddy SR tRNAscan-SE: a program for improved detection of transfer RNA genes in genomic sequence Nucleic Acids Res 1997 25 5 0955 64 10.1093/nar/25.5.0955 PMC146525 9023104

[B32] Bland C Ramsey TL Sabree F Lowe M Brown K Kyrpides NC CRISPR recognition tool (CRT): a tool for automatic detection of clustered regularly interspaced palindromic repeats BMC Bioinformatics 2007 8 1 209 10.1186/1471-2105-8-209 17577412 PMC1924867

[B33] Odermatt A Suter H Krapf R Solioz M An ATPase operon involved in copper resistance by Enterococcus hirae Ann NY Acad Sci 1992 671 484 10.1111/j.1749-6632.1992.tb43836.x 1288347

[B34] Latorre M Olivares F Reyes-Jara A López G González M CutC is induced late during copper exposure and can modify intracellular copper content in *Enterococcus faecalis* Biochem Bioph Res Co 2011 406 4 633 7 10.1016/j.bbrc.2011.02.109 21362400

[B35] Grant JR Arantes AS Stothard P Comparing thousands of circular genomes using the CGView Comparison Tool BMC Genomics 2012 13 1 202 10.1186/1471-2164-13-202 22621371 PMC3469350

[B36] Hasman H Aarestrup FM *tcrB*, a gene conferring transferable copper resistance in Enterococcus faecium: occurrence, transferability, and linkage to macrolide and glycopeptide resistance Antimicrob Agents Chemother 2002 46 5 1410 6 10.1128/AAC.46.5.1410-1416.2002 11959576 PMC127162

[B37] Amachawadi RG Shelton NW Jacob ME Shi X Narayanan SK Zurek L Occurrence of *tcrB*, a transferable copper resistance gene, in fecal enterococci of swine Foodborne Pathog Dis 2010 7 9 1089 97 10.1089/fpd.2010.0540 20500052

[B38] Woese CR Kandler O Wheelis ML Towards a natural system of organisms: proposal for the domains *Archaea*, *Bacteria*, and *Eucarya* Proc Natl Acad Sci 1990 87 12 4576 9 10.1073/pnas.87.12.4576 2112744 PMC54159

[B39] Schleifer K-H. Phylum XIII. Firmicutes Gibbons and Murray. In: *Bergey’s Manual of Systematic Bacteriology*. New York: Springer; 2009: 19–1317.

[B40] Ludwig W, Schleifer K, Whitman W. Class I. Bacilli class nov. *Bergey's Manual of Systematic Bacteriology*. 2009;3:19–20.

[B41] Ludwig W, Schleifer K, Whitman W. Order II. Lactobacillales ord nov *Bergeys Manual of Syst Bacteriol*. 2009;3:463–513.

[B42] Amyes SG Enterococci and streptococci Int J Antimicrob Agents 2007 29 S43 52 10.1016/S0924-8579(07)72177-5 17659211

[B43] Rôças IN Siqueira JF Jr Santos K Association of *Enterococcus faecalis* With Different Forms of Periradicular Diseases J Endod 2004 30 5 315 20 10.1097/00004770-200405000-00004 15107642

[B44] Stuart CH Schwartz SA Beeson TJ Owatz CB *Enterococcus faecalis*: Its role in root canal treatment failure and current concepts in retreatment J Endod 2006 32 2 93 8 10.1016/j.joen.2005.10.049 16427453

[B45] Ashburner M Ball CA Blake JA Botstein D Butler H Cherry JM Gene Ontology: tool for the unification of biology Nat Genet 2000 25 1 25 9 10.1038/75556 10802651 PMC3037419

